# Epidemiological overlaps in COVID-19 and malaria within healthcare and community settings of Southern Ghana

**DOI:** 10.3389/fpubh.2024.1367586

**Published:** 2024-10-01

**Authors:** Gloria Amegatcher, Maame E. Acquah, Deborah K. Tetteh, Rachael Obeng, Ethel Debrah, Bridget Quist, Priscilla Acquah-Jackson, Kyerewaa A. Boateng, Gideon Twieku, Samuel Armoo, Gordon A. Awandare, Lydia Mosi, Charles A. Narh

**Affiliations:** ^1^West African Centre for Cell Biology of Infectious Pathogens (WACBIP), Department of Biochemistry, Cell and Molecular Biology, University of Ghana, Accra, Ghana; ^2^Department of Medical Laboratory Sciences, University of Ghana, Accra, Ghana; ^3^Biomedical and Public Health Research Unit, Council for Scientific and Industrial Research – Water Research Institute, Accra, Ghana; ^4^School of Medicine, Deakin University, Geelong, VIC, Australia; ^5^Department of Medicine, University of Melbourne, Parkville, VIC, Australia

**Keywords:** malaria, COVID-19, diagnostics, co-surveillance, epidemiological overlap, Ghana, community and healthcare

## Abstract

**Background:**

COVID-19 disruptions including lockdowns and prioritization of COVID-19 control programs in Africa in 2020–2022 contributed to reductions in malaria control activities including malaria diagnosis, treatment and resistance monitoring. This study investigated the malaria burden and distribution on the background of active transmission of SARS-CoV-2 in Southern Ghana; utilizing community health education and medical screening (CHEMS) approach to determine epidemiological overlaps in COVID-19 and malaria.

**Methods:**

Between October–December 2022, prospective cross-sectional surveys, with CHEMS were conducted in Greater Accra and Central regions, where 994 participants enrolled either at a hospital or community setting provided demographic and clinical data including history of clinical malaria infection and antimalarial treatment in the past 2 weeks. Of this study population, 953 provided nasal/throat swabs for COVID-19 RT-PCR testing, with a subset of 136 participants also providing finger-prick blood for malaria RDT testing.

**Results:**

The study population comprised of 73.6% adults, with 54.6% COVID-19 vaccination rate. Overall, 18.1% of participants had a history of clinical malaria, which was associated (adjusted odds ratio > 1.50, *p*-value ≤0.022) with COVID-19 symptoms and positivity, study area and hospital setting, suggestive of overlaps in the epidemiological risk for malaria. On a background of widespread SARS-CoV-2 infections (12–37%), malaria parasitaemia was detected in 6%, with 2% being co-infections with SARS-CoV-2. Among the malaria positives, 9.5% had a history of antimalarial treatment, which suggested that their infections were recrudescent parasitaemia.

**Conclusion:**

The epidemiological and clinical overlap between malaria and COVID-19 within the hospital and community settings underscores the need for accurate case diagnosis to inform effective clinical treatments. Innovative surveillance programs, with community engagement are needed to maximize control interventions including treatment of asymptomatic malaria infections.

## Introduction

COVID-19 disruptions accounted for 14 million increase in malaria cases in the sub-Sahara Africa (SSA) region in 2020 (1, 2) indicating that innovative control programs including diagnosis and treatment and their delivery systems are needed to supplement the activities of National Malaria Control/Elimination Programs (NMCPs or NMEPs) (3). Malaria remains a major public health threat in SSA, with children under 5 years and pregnant women being the most vulnerable (1). Therefore, it is important that control programs remain active.

Malaria surveillance in Ghana, mostly focused on clinical infections, was disrupted by the COVID-19 pandemic. For instance, there were increased cases of malaria after the first wave of COVID-19 in March 2020 (4), with low treatment-seeking at health facilities (4). Additionally, other reports showed a decline in the uptake of mass test, treat and track (MTTT) of malaria in some rural communities in Ghana (5). Public engagement in infectious diseases surveillance in Ghana remains a challenge for control programs (6). Community Health Education and Medical Screening (CHEMS), an incentivized approach to community participation and engagement in public health, implemented by the University of Ghana to sensitize communities to COVID-19 interventions (7), can provide synergy for malaria surveillance programs at the national level.

Indeed, significant overlaps in the clinical presentations and epidemiological distribution of malaria, caused by *Plasmodium falciparum*, and COVID-19 cases in endemic communities in Ghana have contributed to malaria misdiagnosis and impacted the effectiveness of interventions (8). While uptake of interventions at the community level declined during the COVID-19 disruptions (5), there is limited data to assess malaria case and co-infection rates in communities with active SARS-CoV-2 transmission.

Our team comprising of researchers and health personnel were actively involved in several national COVID-19 response initiatives between 2020 and 2023, leveraging programs including CHEMS to diagnose and track SARS-CoV-2 transmission networks (7, 9, 10). Therefore, amidst the limited resources and disruptions in malaria control programs, we tested the feasibility of integrating malaria surveillance into these COVID-19 initiatives, with the aim of determining the distribution of the disease and its epidemiological overlaps with COVID-19, and additionally, to explore ways of expanding test/treatment services in Southern Ghana.

## Methods

### Study design

**Figure 1:**
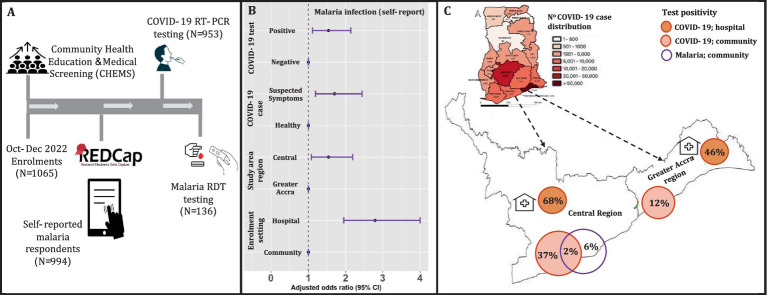
Enrolment with COVID-19 and malaria testing in the Greater Accra and Central regions of Ghana. **(A)** Enrolment, CHEMS and testing for SARS-CoV-2 (both study areas) using throat and/or nasal swabs, and malaria RDT testing using finger-prick blood (only Central region). **(B)** The adjusted odds ratio for reported malaria in the past 2 weeks. These are the results of the logistic regression with self-reported malaria infection as the outcome, with age and gender as covariates in the final regression. The unadjusted regression produced qualitatively similar effect sizes. The reference variables are indicated with odds ratio of 1. **(C)** The map of Ghana shows cumulative COVID-19 case distribution in all 16 regions based on routine surveillance data made publicly available by the Ghana Health Services (17). The extended map shows the COVID-19 and confirmed malaria infection rates for the study areas: Greater Accra and Central regions. The malaria infection rate for this study was assessed only in the study communities in the Central region.

This study was embedded within a COVID-19 pre-clinical study that aimed to assess the clinical performance of a novel point-of-care SARS-CoV-2 RNA test. The details of the pre-clinical study have been described elsewhere (Trial Registry # ACTRN12623000066684). Briefly, between October–December 2022 (Figure 1A), 1,065 participants were recruited through multi-site prospective cross-sectional surveys in eight communities in Greater Accra and four communities in the Central regions of Ghana. The feasibility of the CHEMS approach was thus tested and incorporated as pivotal to community acceptance and engagement in the study.

### Enrolment

In each community where and when possible, the participants were recruited through convenience sampling at hospitals/clinics (hospital setting) and at public places (community setting) including markets and schools (Figure 1A) and enrolled onto the parent COVID-19 study as well as the malaria study presented in this paper. Through CHEMS, the field staff educated the community and raised awareness about COVID-19 testing and vaccination, malaria diagnosis and treatment, and clinical symptoms of both diseases. Additionally, participants were provided with free medical screening including body temperature and weight checks and free private consultation with the study nurse. All participants provided informed consent for study participation and sample collection.

### Sample collection and testing

Eligible participants, all ages with/without respiratory disease including COVID-19 symptoms (headache, fever, cough, etc) provided nasal/throat swabs (Figure 1A) for COVID-19 RT-PCR testing using the two commercial kits: 2019-nCoV kit, Sansure Biotech and Allplex^™^ SARS-CoV-2 Master Assay, Seegene. The test results of the novel point-of-care test were used to confirm SARS-CoV-2 infection in samples with invalid RT-PCR test results. Participants in the Central region also had the option of providing finger-prick blood for malaria RDT testing using the Bioline^™^ Malaria Ag P.f test (Abbot) (Figure 1A). The latter was due to limited malaria RDT test availability for this study.

### Data collection

The participants’ demographic and clinical data including self-reported malaria infection and antimalarial treatment in the past 2 weeks were recorded in a Research Electronic Data Capture software (REDCap) (Figure 1A). Using REDCap provided the advantage of storing data online and offline and tracking information in real-time as well as being able to record the locations where participants were enrolled via GPS. From the participants, data was also collected on symptoms to understand if malaria and/or COVID-19 cases were symptomatic or asymptomatic. Where available, positive cases (malaria or COVID-19) were referred to the nearest hospital/clinic for further confirmation and clinical treatment.

### Data analysis

For this study, participants were categorized into age groups: <18 (children), 18–59 (adults) and ≥ 60 (older adult); Statistical analyses including comparison of proportions and logistic regression were performed as described elsewhere (11) using R v3.5.2 and STATA SE 18. Chi-square or Fisher’s (*n* ≤ 5) exact test were used to compare proportions. Holm-Bonferroni method was used to adjust for multiple testing. We then performed logistic regression [cluster variance (VCE) method] to determine the risk (odds ratio or OR) of participants, under the study variables, to self-report having malaria in the past 2 weeks. Age and gender were considered potential confounders and were adjusted for in the final regression. The adjusted odds ratios with 95% confidence interval (CI) were plotted with the ggplot2 R package. Statistical significance; *p*-value <0.05.

## Results

### Demographics

Of the 1,065 eligible participants enrolled, 994 responded to questions on malaria infection, and therefore they constituted the final study population. Of this, 953 provided swabs for COVID-19 testing and 136 provide finger-prick blood for malaria testing (Figure 1A). The demographics of the study population is shown in Table 1, focusing on self-reported malaria infection stratified by the study variables. The majority of the study population comprised of adults (73.6%) and females (61.1%). Based on COVID-19 case definition (12), 74.9% of the study participants reported having no clinical symptoms associated with COVID-19 (hereafter referred to as “healthy participants”) and 23.1% reported having COVID-like symptoms (“suspected cases”).

**Table 1:** Malaria infection in the study population in the Central region of Ghana.

Demographic characteristics		Self-reported malaria (*N* = 994)			Malaria RDT testing (*N* = 136)		
		Total (N)	YES (%)	*p*-value	Total (N)	Positivity (%)	*p*-value
Age groups	Children	197	17.8	0.988	82	2.4	0.102
	Adults	732	18.2		47	10.6	
	Older adult	65	18.5		7	14.3	
Gender	Male	384	20.3	0.187	42	4.8	0.700
	Female	602	16.8		93	6.5	
Catchment area (Region)	Greater Accra	365	14.2	0.020	0	0.0	NA
	Central	629	20.3		136	5.9	
Enrolment setting	Community	619	12.9	0.001	136	5.9	NA
	Hospital/Clinic	375	26.7		0	0.0	
COVID-19 vaccination	Vaccinated	531	16.4	0.105	40	7.5	0.429
	Unvaccinated	441	20.6		93	4.3	
Had COVID-19 past 1-month	YES	80	17.5	0.920	24	4.2	0.680
	NO	874	18.0		110	6.4	
COVID-19 case	Suspected	231	25.5	0.003	17	0.0	0.595
	Healthy	688	16.4		118	6.8	
Confirmed COVID-19	Positive	373	22.8	0.022	54	1.9	0.211
	Negative	580	15.0		82	8.5	
Malaria treatment	Clinical treatment^#^	125	99.2	0.520	21	9.5	1.000
	Self-medication*	52	100.0		5	100.0	

Not applicable (NA) as malaria RDT testing was only completed in the community setting in the Central region only. *Self-medication includes participants who reported non-antimalarial treatment and no treatment. ^#^Clinical treatment includes participants who reported being given approved antimalarials at the hospital or pharmacy. The *p*-values in the table were calculated as part test of proportions using the chi-square/fisher test. That is, a test of statistical significance difference in the proportion. For analysis under some study variables, participants with missing data for that variable were excluded from the analysis and therefore, the subtotal is < 994.

### COVID-19 confirmation

COVID-19 case confirmation by RT-PCR testing resulted in 39% positivity in the study population. At the community and hospital settings, 12 and 46%, respectively, were recorded for Greater Accra, and 37 and 68%, respectively, for the Central region (Figure 1C). The COVID-19 vaccination rate overall was 54.5, and 91.6% of the participants reported not having COVID-19 in the last 1 month (Table 1).

### Association between COVID-19 and self-reported malaria

Next, we determined the association between malaria infection (self-reported), in the previous 2 weeks, and epidemiological factors including COVID-19 disease. Overall, 18.1% of participants reported having clinical malaria (Table 1), of which 70.6% received clinical treatment (antimalarials from the hospital or pharmacy). Self-medication included the use of non-antimalarials that were not clinically prescribed (hospital or pharmacy). Self-reported clinical malaria was significantly associated (adjusted odds ratio > 1.50, *p*-value ≤0.022) with COVID-19 (suspected symptoms and test positivity), enrolment setting (hospital/clinic) and study area (Central region) (Figure 1B). These results showed that epidemiological (e.g., health settings) and/or clinical (clinical disease/signs) risk factors associated with COVID-19 overlapped with malaria in our study communities.

### Confirmation of *Plasmodium falciparum* and SARS-CoV-2 co-infections

We then conducted malaria RDT testing in the Central region, which had the highest risk of self-reported malaria. Confirmation of *P. falciparum* infections among the subset of 136 participants (60.3% being children) resulted in a parasite prevalence of 6.0% (Figure 1C); The majority of those tested being adults with no history of clinically treated malaria in the previous 2 weeks, suggesting that the current infections were recently acquired. A minority of those tested, 9.5%, had history of clinically treated malaria in the previous 2 weeks, suggesting the current infections were recrudescent parasitaemia or newly acquired infections. Interestingly, 2.0% of the confirmed malaria positive cases were co-infected with SARS-CoV-2 (Figure 1C); They were children with no previous history of COVID-19 or malaria in the previous 2 weeks. Co-infections of *P. falciparum* and SARS-CoV-2 could have impacted clinical/home management of both diseases in our study communities.

## Discussion

Routine malaria surveillance in endemic communities is crucial to inform and assess the effectiveness of control interventions including diagnosis and antimalarial treatment. These interventions were considerably disrupted in many parts of Ghana as a result of the COVID-19 epidemics (5). Therefore, this study evaluated the malaria burden and its epidemiological overlaps with COVID-19 in Southern Ghana.

Our findings in the Greater Accra and Central regions in October–December 2022, based on the self-reports, suggested that malaria was moderately prevalent, and varied heterogeneously, from 13% in the community setting to 27% in hospital settings. Among the reported cases, ~71% reported using clinically prescribed antimalarials, likely representing high treatment-seeking in healthcare facilities (5); Indeed, we observed that participants enrolled in a hospital/clinic setting were up to 4 times more likely to report having malaria in the past 2 weeks compared to those enrolled in the community, i.e., public places. It is unlikely that this treatment-seeking was due to COVID-19 since the majority, 91.6%, had no history of COVID-19 in the last 1 month. Outpatient data collected during the COVID-19 epidemics in Ghana showed that treatment-seeking due to malaria increased in communities in the south (5) compared to the north (4). This uptick in treatment-seeking happened post-lockdowns and following rollout of COVID-19 vaccination programs in early 2022 (5).

The high rate of self-reported malaria in the Central region (20.3%) compared to the Greater Accra (14.2%) region is consistent with the 2022 malaria parasite prevalence data (10–23% in the Central region versus 5–15% in Greater Accra) (13), and corroborates similar findings in the Eastern region where there was low uptake of mass test, treat and track of malaria due to COVID-19 disruptions (5). Further studies are needed to assess whether self-reported malaria data may be informative for surveillance programs including targeted control interventions in the two regions. Example, the Greater Accra and Central regions, respectively, are located within the low transmission areas (1–199 cases per 1,000 population or 1–5% parasite prevalence) and moderate transmission areas (200–499 cases per 1,000 population or 5–15% parasite prevalence) (13). These stratifications have informed the activities of the NMEP’s 2024–20287 strategic plan, with mass drug administration (MDA) and seasonal malaria chemoprevention (SMC) being earmarked for the two regions, respectively (13).

Malaria parasite prevalence in the study communities in the Central region was 6%, comparable to the 6.8% prevalence (range 4–11%) that we estimated from a study of afebrile malaria cases in four communities located in the same region (14). Aside the self-reported malaria and COVID-19 data we collected from the participants, we also conducted RT-PCR for COVID-19 and RDT for malaria, which revealed a high asymptomatic infection rate. The asymptomatic outcomes observed were among healthy adults, which is not unusual in high transmission settings like Ghana where the majority of malaria cases are asymptomatic (15), and have been shown to contribute to the transmission of drug resistance malaria (16). Additionally, our data suggest that the asymptomatic malaria infections we detected were recent infections or chronic parasitaemia. However, we cannot rule out the possibility that they were recrudescent infections, particularly, among the RDT-positive cases with a history of clinically treated malaria in the past 2 weeks. Although clinical resistance to artemisinin combination therapies (ACTs), the standard treatment of care for uncomplicated malaria, has not been reported in Ghana (16), our study highlights the need to monitor and prioritize asymptomatic malaria infections during interventions, and to confirm cases with accurate diagnostic tests.

Our study demonstrates the feasibility of using CHEMS to conduct both malaria and COVID-19 surveillance in communities and health settings with active malaria and COVID-19 transmission. This allowed us to maximize the limited resources at our disposal; Indeed, during the pandemic there was reallocation of resources, including health service systems/personnel and funding, from other diseases such as malaria to COVID-19 to help public control efforts of the latter (5, 8). Therefore, by utilizing the CHEMS approach this study was able to conduct co-surveillance of COVID-19 and malaria and helped identified risk factors of infection to inform control of both diseases. Additionally, our study findings also underscore the relevance for continuous malaria surveillance in both clinical and asymptomatic cases in low-resource settings of Southern Ghana.

By integrating malaria surveillance into COVID-19 programs in Ghana, we uncovered epidemiological overlaps between the two diseases in our study communities. Our data suggest that epidemiological (e.g., health settings) and/or clinical (e.g., symptoms) risk factors associated with COVID-19 overlapped with malaria. The majority of the SARS-CoV-2 and *P. falciparum* infections were asymptomatic, and mostly in the adults, with 2% being co-infections. It is well known that malaria and COVID-19 could have similar clinical presentations (e.g., fever, headache, fatigue) and as such presumptive diagnosis based on clinical signs could impact clinical management. Therefore, our study highlights the need for definitive diagnosis based on laboratory test confirmations.

### Limitations and recommendations

The sample size for the RDT positive malaria cases was insufficient to detect any statistically significant association between *P. falciparum* and SARS-CoV-2 infections. Therefore, further investigations with larger sample sets are needed to evaluate the interplay between both diseases, within different epidemiological and geographical settings, and how control interventions impact the broader disease distribution require investigations. The co-surveillance of malaria and COVID-19 needs to be tested in other malaria endemic regions, including areas with low *P. falciparum* transmission.

## Conclusion

In summary, the epidemiological and clinical overlap between malaria and asymptomatic COVID-19 within the hospital and community settings underscores the need for accurate case diagnosis to inform effective clinical treatments. The CHEMS approach allowed us, researchers and clinicians, to effectively engage with the community, maximizing the limited resources at our disposal to conduct co-surveillance activities for COVID-19 and malaria in Southern Ghana.

## Data Availability

The original contributions presented in the study are included in the article/supplementary material, further inquiries can be directed to the corresponding author.
